# Knowledge and Attitude of Iranian Youth Toward AIDS: A Systematic Review and Meta-Analysis in Iran

**DOI:** 10.25122/jml-2019-0087

**Published:** 2020

**Authors:** Victoria Momenabadi, Maryam Safarnavadeh, Fatemeh Nazari Robati, Seyed Masoud Mousavi, Elham Nejadsadeghi, Gholamreza Masuody, Leili Moayed, Maryam Seraji

**Affiliations:** 1. Kerman University of Medical Sciences, Kerman, Iran; 2. Ministry of Health and Medical Education, Tehran, Iran; 3. Social Determinants of Health Research Center, Institute for Futures Studies in Health, Kerman University of Medical Science, Kerman, Iran; 4. Neuroscience Research Center, Kerman University of Medical Science, Kerman, Iran; 5. School of Public Health, Behbahan University of Medical Sciences, Behbahan, Iran; 6. Health Promotion Research Center, Zahedan University of Medical Sciences, Zahedan, Iran; 7. Iranian Research Center on Healthy Aging, Sabzevar University of Medical Sciences, Sabzevar, Iran

**Keywords:** AIDS, attitude, youth, Iranian, knowledge, systematic review

## Abstract

Inadequate knowledge and negative attitudes are the major hindrances to prevent the spread of the human immunodeficiency virus. This study aims to assess the knowledge and attitude toward the human immunodeficiency virus and acquired immune deficiency syndrome among youths in Iran.

We conducted a systematic review, searching online databases until July 2018, focusing on knowledge and attitudes about the human immunodeficiency virus and acquired immune deficiency syndrome in Iranian youths. We included the studies that aimed to determine the knowledge and attitudes of people from Iran and were conducted in the last 18 years.

In total, 14 eligible papers (out of 300) were entered into the analysis, and the overall knowledge of Iranian youth toward the acquired immune deficiency syndrome was 57.6% (95% CI: 56.7%-58.5%). Also, the results of Cochran’s test showed the heterogeneity of the studies (Q=1578.2, df=13, I2=79.4%, p<0.001).

We concluded that our results would guide the development of population-focused knowledge and attitude about the human immunodeficiency virus and acquired immune deficiency syndrome in Iran, which is lacking among the general public and healthcare staff.

## Context

The acquired immunodeficiency syndrome (AIDS) is a fatal retroviral disease that damages the human immune system and makes its victim vulnerable to opportunistic infections [1]. With about three decades of AIDS, this infectious disease has become a global problem and threatens the health of people all over the world [2]. AIDS is the fourth cause of death and the second most infectious disease worldwide [3, 4]. Although the incidence of infection with the human immunodeficiency virus (HIV) has stabilized in many developed regions of the world, the number of infected people is increasing in other parts of the world, especially in Africa, Southeast Asia, and Eastern Europe [5]. According to the World Health Organization (WHO), between 16,000 to 20,000 people are infected with HIV daily, and nearly 3 million people die each year [6]. According to estimates reported by the WHO, at the end of 2011, 34 million people were infected with HIV worldwide, of which about 50% are women and 10% are children [7]. Young people, the world’s largest group of people living with AIDS, face the risks of unsafe sexual experiences and drug use due to their curiosity, friends’ pressure and lack of knowledge and skills; therefore, these groups are more at risk for HIV and AIDS compared to adults [8]. According to the global statistics, five thousand young people are infected with HIV every day, 6000 cases per minute, and the most common way of infecting young people is through sexual intercourse [9-12].

Since HIV/AIDS affects mainly the young, the prevention of HIV spread requires the implementation of ongoing and targeted educational programs for this age group because of the specific circumstances of the population pyramid, which consists of about 15-50% of the Iranian population [12-13].

There is no definitive treatment for AIDS, and the only way to fight this disease is to prevent the population from getting infected because the virus is often acquired through avoidable and modifiable behaviors [10]. So, the most crucial barrier to AIDS prevention is the lack of awareness of various aspects of the disease [2], and education and information can be considered as appropriate strategies to prevent new cases of infection. Studies in Iran and different countries show that young people’s levels of awareness about HIV/AIDS are often different and often inadequate due to several factors such as society, culture, adherence to religious issues, political policies, and parental awareness. Young people’s awareness of HIV transmission and sex drive is critical because an individual’s knowledge and attitude affect their behavior [2]. The purpose of this study is to systematically review the awareness and attitude of Iranian youths about AIDS.

## Evidence Acquisition

This systematic review was designed in 2016 and commenced in accordance with the PRISMA (Preferred Reporting Items for Systematic Reviews and Meta-Analyses) guidelines [14].

### Search Strategy

A literature search of published studies was carried out using international databases (Medline/PubMed, Scopus, Web of Sciences, Embase, and Google Scholar) for English papers and Iranian databases (Scientific Information Database) (www.sid.ir), MagIran (www.magiran.com), IranMedex (http://www.barakatkns.com), and Irandoc (www.irandoc.ac.ir)) for Persian papers. The Medical Subject Headings (MeSH) keywords were used, including “Knowledge”, “Attitudes”, “Youth”, “HIV” and “Iran”. The obtained papers were imported into an EndNote X7 library (Thomson Reuters, Carlsbad, CA, USA), and duplicates were removed. No language or time limitations were considered.

### Selection Criteria and Quality Assessment

Two reviewers independently reviewed all resultant titles and abstracts. The full-texts of all relevant articles were then assessed by each reviewer. Original studies in English or Persian that provided a study of the youth knowledge and attitude about AIDS in young people were included in this systematic review and prospective, case-control, experimental studies, clinical trials, reviews, commentaries, case reports, systematic reviews, and meta-analyses were included. One of the researchers extracted the data from all eligible studies and entered them into a pre-designed data extraction form. The study setting and design and youth knowledge and attitude about AIDS in youths were extracted. Then, the results were synthesized in a narrative review. Discrepancies between the reviewers were resolved by consensus.

### Quality Assessment

In order to assess the quality of articles, a checklist prepared by the Joanna Briggs Institute (JBI) was used [15]. The purpose of this appraisal is to assess the methodological quality of a study and determine the extent to which a study has addressed the possibility of bias in its design, conduct, and analysis.

### Risk of Bias Across Studies

A random-effects model was used for minimizing the risk of bias across studies [16, 17].

## Statistical Analysis

Heterogeneity was assessed by Cochran’s Q test (with a significance level of p≤0.1) combined with an I^2^ index (with a significance level of I^2^ >50%). In the presence of significant heterogeneity (p≤0.1 and I^2^≥50%), the random effect model (with inverse variance method) was utilized. In the case of no evidence of heterogeneity (p>0.1 and I^2^<50%), the fixed effect model was employed. All analyses were carried out using the Stata software, version 12 (Stata Corp LP, College Station, Texas).

## Results

**Figure 1:**
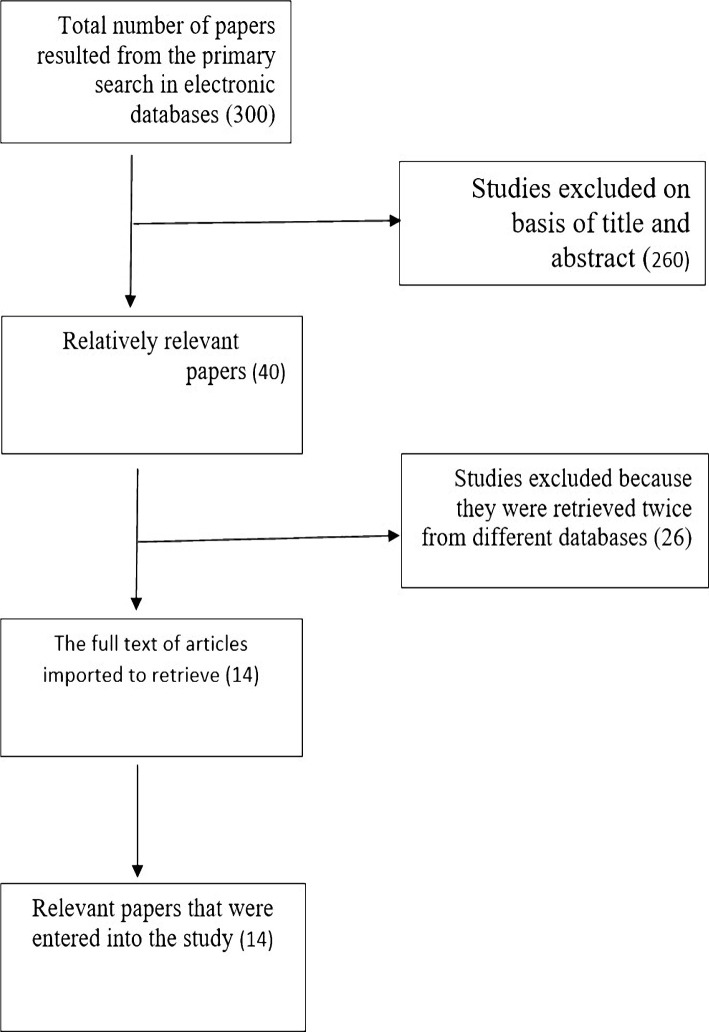
Flowchart of selection of the studies included in the present systematic review on the AIDS-related high-risk behaviors in youth.

Totally, 300 titles were gathered from PubMed, Web of Science, Scopus, Science Direct, Google Scholar, SID, and Magiran databases. Further screening for the relevant articles based on title and abstract yielded 40 articles. However, 26 articles were excluded after reviewing and applying the inclusion criteria. Hence, 14 studies were eligible for systematic review. The flowchart of the study has been depicted in Figure 1.

Characteristics of published articles by Iranian researchers on online databases through July 2018 have been listed in Table 1.

**Table 1:** Characteristics of published articles by Iranian researchers on online databases until July 2018.

**Authors**	**Years**	**Sample size**	**The most important findings**
Dadkhah et al. (18)	2008	400	Only 67.5% of the participants had a moderate knowledge score. Negative levels of attitude were observed among 25.5% of the participants.
Abdeyazdan and Sadeghi (21)	2003	350	The total knowledge level of 60.2% of students was good; 34.1% of them had moderate and 5.7% had poor knowledge levels; In 68.6% of students the attitude was moderate, in 23.3% the attitude was good and in 8.1% of students the attitude was poor.
Movahed and Shoaa (33)	2008	600	The total knowledge level of 18.8% of students was high, 36.9% had moderate and 44.3% of the subjects had low knowledge levels; In 69.8% of students, the attitude was moderate, in 15.4% the attitude was positive and in 14.9% of students the attitude was negative.
Tavoosi et al. (22)	2002	4641	67 % of the students had good knowledge.
Ghabili et al. (23)	2005	300	Tabrizian female students have overall negative attitudes towards HIV/AIDS.
Yazdi-Ravandi et al. (24)	2015	509	The overall rate of knowledge and attitude about HIV/AIDS among students of the Hamadan University of Medical Sciences was acceptable.
Rahmati Najarkolaei et al. (11)	2009	664	92% of the population had good awareness of AIDS.
Alipour et al. (25)	2012	588	Students had high levels of knowledge and attitudes about HIV/AIDS, but this level of awareness was not enough, and students were in need of extensive training programs in this field.
Vahdaninia and Mozafarzadeh (26)	2009	581	Girls' highschool students had a fairly good knowledge of AIDS and had a positive attitude toward AIDS patients.
Fadaei et al. (27)	2007	500	47.6% of students had good knowledge and 39.6% had moderate awareness, 48% of students had a poor attitude and 19% had a good attitude.
Rohani rad and Kolahi (28)	2006	655	Public awareness of 45.5% of the students was good, 45.2 % moderate and 9.3% poor.
Azimian (29)	2005	100	The highest level of awareness among students working on a PhD level and the lowest level among laboratory sciences.
Karimi et al. (34)	2000	1850	23.2% of the students had very poor knowledge, 35% had poor knowledge, 34.4 % had moderate and 5.4% good knowledge and only 3% of students had perfect information.
Ranjbar (30)	2009	800	474 cases (59.2%) had good knowledge, 312 cases (39%) had an intermediate level, 14 cases (18%) had poor knowledge, 371 cases (46.4%) had a favorable attitude and the rest had a neutral and negative attitude, respectively.
Panahande and Taramian (31)	2004	850	11.7% of the students had good knowledge, 77.6% moderate and 10.7% had poor knowledge.
Marashi et al. (35)	2000		The mean knowledge score was 20.28±0.68 and the mean attitude score was 66.13±2.78; the majority of students (88.5%) had high levels and (11.5%) had a moderate knowledge level. 88.75% had a positive attitude, 11.2% had good awareness. 52.4% of subjects had a negative attitude towards HIV transmission.
Sanei Moqadam et al. (32)	2010	951	50.2% of students had good, 44% moderate and 5.8% poor knowledge. 17.1% with positive attitude, 56.9% neutral attitude and 25.9 % negative attitude
Ramazan Khani et al. (19)	2010	590	50.3% of the study population had low level, 36.9% had an average level, and 12.7% had a high knowledge level. Attitude ranking was low in 13.7%, moderate in 62.2% and low in 23.7%.
Lotfipour Rafsanjan (8)	2011	384	Mean scores of knowledge was 50.4±4; mean scores of attitude was 18.4±2.26. 89.6% of subjects had good knowledge, 80.2% had a relatively positive attitude.

The results of knowledge studies were evaluated by assessing the mean score of knowledge, awareness, percentage and ranking. The reporting level was fluctuating, and most studies have indicated a low level of awareness (Table 1).

The results of studies on women’s attitudes toward AIDS in the reviewed literature showed that reported attitudes as an average point of attitude, and the attitude of the ranking were reported.

In most studies, positive and negative points of view were ranked. The evaluation tool for assessing awareness was a researcher-made questionnaire with a number of different questions.

**Figure 2:**
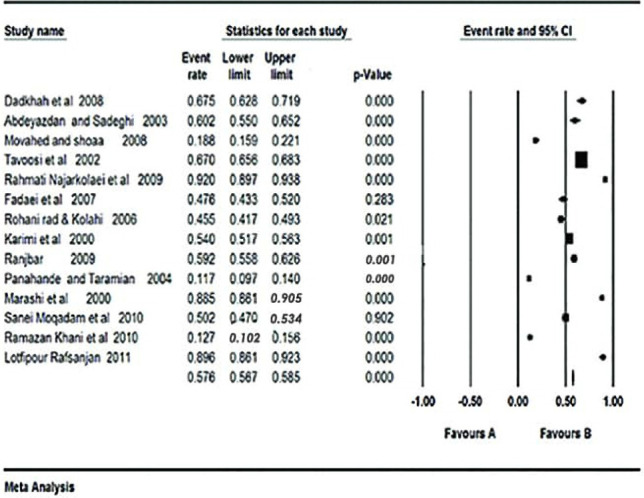
Forest plot of the random-effect meta-analysis for knowledge of Iranian youth toward AIDS.

In total, 14 eligible papers (out of 300) were entered into the analysis. The overall knowledge of Iranian youth toward AIDS was 57.6% (95% CI: 56.7%-58.5%). The results of the Cochran’s test showed the heterogeneity of studies (Q=1578.2, df=13, I2=79.4%, p<0.001) (Figure 2).

## Conclusions

In this study, 20 articles were studied on the youths’ awareness and attitudes about AIDS. The articles reported different levels of knowledge about AIDS. Dadkhah et al. showed that first-year college students’ knowledge and attitudes were about 67.5% and 25.5%, respectively [21]. Ramazan Khani et al. conducted a study in 2010 on 590 subjects from 15 to 25 years old and showed that the level of awareness about AIDS was lower than the average, especially in people with high-risk sexual behavior [22]. Ayranci, in an epidemiological study conducted in Turkey, reported a relatively good level of knowledge, which had a statistically significant relationship with the level of education. The social attitude and social support were positive for AIDS patients [23]. The results of these studies and other studies indicate the need to improve knowledge and attitude towards AIDS. In these studies, there was a wide range of attitudes toward AIDS. In a study by Lotfipour et al. from 2011, students’ attitude toward AIDS was positive [11].

In the reviewed articles, young people’s awareness and attitudes toward AIDS were varied, and these differences could be related to the inconsistency of research tools and questionnaires, the content of the questions, the number of questions, sample size, the methodology, and the type of study. Therefore, it is necessary to study the level of awareness and attitude towards AIDS using a comprehensive questionnaire designed according to cultural and social conditions.

Today, AIDS awareness is good, but the outbreak of the disease and its related behavior show that only effective education can lead to a change in attitude and behavior. Also, training should provide skills to prevent high-risk behaviors. If a person’s knowledge cannot provide preventive skills for high-risk behaviors, it does not reduce the incidence of this disease.

### Limitation of the study

The current study had several limitations, and the first was publication bias. We attempted to minimize this problem by searching various databases in English and Persian, but we did not have access to non-published articles. Second, the studies were performed in different parts of the country, and cultural differences might have affected the study. However, this issue was not addressed in the selected studies.

### Recommendation

Future systematic reviews and meta-analyses investigating the knowledge and attitude of Iranian youth toward AIDS should not be limited to Iran, and areas covered by the WHO should be taken into consideration as well.

## Acknowledgments

The authors would like to thank Ms. Seyedeh Leila Dehghani from the Department of Public Health of the Behbahan Faculty of Medical Sciences for her assistance in editing this manuscript.

## Conflict of interest

The authors declare that there is no conflict of interest.
